# Anaesthesia Challenges in a Neonate Having Supracardiac Total Anomalous Pulmonary Venous Return With Incidental Stridor for Non-operating Room Anaesthesia (NORA): Feed and Wrap Technique as a Rescue

**DOI:** 10.7759/cureus.30357

**Published:** 2022-10-16

**Authors:** Ajay Singh, Kashish Garg, Venkata Ganesh, Naveen Naik B, Damandeep Singh

**Affiliations:** 1 Anaesthesiology, Post Graduate Institute of Medical Education and Research (PGIMER), Chandigarh, IND; 2 Anaesthesia and Intensive Care, Post Graduate Institute of Medical Education and Research (PGIMER), Chandigarh, IND; 3 Radiodiagnosis, Post Graduate Institute of Medical Education and Research (PGIMER), Chandigarh, IND

**Keywords:** total anomalous pulmonary venous return, airway anomaly, feed and wrap technique, non-operating room anaesthesia, neonate

## Abstract

This case represents anaesthetic challenges while managing a neonate having a congenital cardiac anomaly with incidental stridor for the non-operating room anaesthesia (NORA). Anaesthesia management of neonates is demanding even for experienced anaesthetists in terms of vascular access, airway management, relatively poor respiratory reservoir and transitional cardiac physiology. Neonate having cyanotic congenital heart disease with abnormal cardiac physiology demands further attention. In children with congenital heart disease, difficult intubation remains a possibility as they are more often associated with airway anomalies. NORA has its own set of anaesthesia challenges related to a lack of adequate anaesthesia equipment and monitoring devices, a limited and unfamiliar workspace, lack of trained support staff along with more medically complex patients. Recently there has been concern regarding the safety of anaesthetic agents in children < 3 years. So, techniques are being developed to promote immobilisation without using anaesthesia in short procedures. One such technique is the “feed and wrap” technique where feeding and swaddling induce natural sleep in infants. Here, we have used the “feed and wrap technique” in a 24-day-old neonate having supracardiac total anomalous pulmonary venous return with incidental stridor posted for contrast-enhanced computed tomography angiography.

## Introduction

Total anomalous pulmonary venous return (TAPVR) is a rare disorder affecting 0.6 to 1.2 per 10 000 live births which accounts for only 0.7% to 1.5% of children having congenital heart disease (CHD) [1]. Out of this, less than 7% survive into adulthood [1]. Often these children are posted for contrast-enhanced computed tomography (CECT) angiography to know their complex cardiac physiology for further plan of surgical management. Providing sedation to these children in the non-operating room area remain a challenge even for experienced anaesthetist due to their unpredictable response to anaesthetic agents, difficult airway, relatively poor respiratory reserve, difficult vascular access and postoperative anaesthesia complications [2,3]. In children with CHD, difficult intubation remains a possibility as they are more often associated with airway anomalies. Along with this, recent literature raises concerns regarding the safety of anaesthetic agents in children less than three years [4-7]. Non-operating room anaesthesia (NORA) has its own set of anaesthesia challenges related to a lack of adequate anaesthesia equipment and monitoring devices, a limited and unfamiliar workspace, and a lack of trained support staff along with more medically complex patients. So, techniques are being developed to promote immobilisation without using anaesthesia in short procedures, especially in the NORA. One such technique is the “feed and wrap” technique where feeding and swaddling induce natural sleep in infants.

## Case presentation

A 24-day-old full-term neonate with supracardiac TAPVR with incidental stridor was posted for CECT angiography. The child was born full term by lower segment caesarian section because of foetal distress with a body weight of 2.9 kg. The child cried immediately after birth with a bluish body which resolved on oxygen supplementation. The child was kept in the incubator for 24 hrs and then discharged. The child had a history of the suck-rest-suck cycle; however, there was no history of tachypnoea or sweating during feeding. After 10 days, the child came for regular follow-up in the outpatient department, and on examination, the child was found to have a heart rate of 170/min, respiratory rate (RR) of 60/min, pansystolic murmur in the tricuspid area with subcostal retractions, room air oxygen saturation of 80%, and adequate central and peripheral pulses. 2D echocardiography revealed supracardiac TAPVR, pulmonary veins draining into superior vena cava (SVC) at or above the SVC-RA (right atrium) junction, dilated RA and right ventricle (RV), severe pulmonary artery hypertension, moderate tricuspid regurgitation, moderate to severe atrial septal defect (ASD) with the right to left shunt, 3mm patent ductus arteriosus with the left to right shunt, and normal biventricular function. A renal function test before CECT was within normal limits.

**Figure 1:**
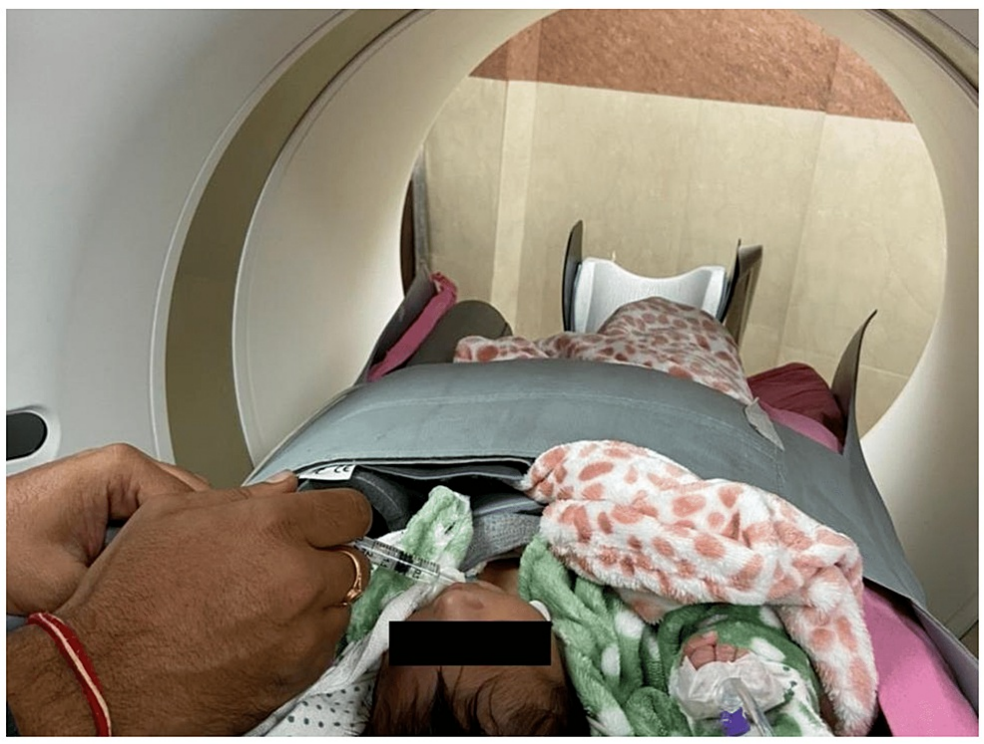
A 24-day-old neonate undergoing contrast-enhanced CT angiography using the “Feed and Wrap” technique.

On the day of CECT, the child's fasting status was confirmed and a preoperative anaesthesia assessment was done. No other comorbidity or recent upper respiratory tract infection was present. On examination, audible stridor was present which was not evaluated earlier. The child had room air oxygen saturation of 81% with RR 64/min. Intravenous access was taken in the right upper limb with a 26 G cannula in the mother’s lap. Electrocardiography and pulse oximeter monitoring was done during CT. A gauze piece soaked with 10% dextrose was put in the baby’s mouth as a pacifier after attaching all monitors to soothe the baby. Subsequent 1mL 10% dextrose aliquots were given as required. The baby was swaddled in a blanket secured with elastic velcro bandages so the child could not move. Oxygen supplementation was given through the tubing placed slightly below the chin. No anaesthetic drug was given during the procedure as the child had an unknown cause of stridor along with complex cardiac physiology. The child's vitals remained stable during the procedure. With this technique, we were able to obtain a good quality image of CECT angiography which revealed all pulmonary veins draining into a common channel measuring 7 mm which further drains into SVC making a diagnosis of supracardiac TAPVR with dilated RA, RV and ASD of 1.1 cm.

**Figure 2:**
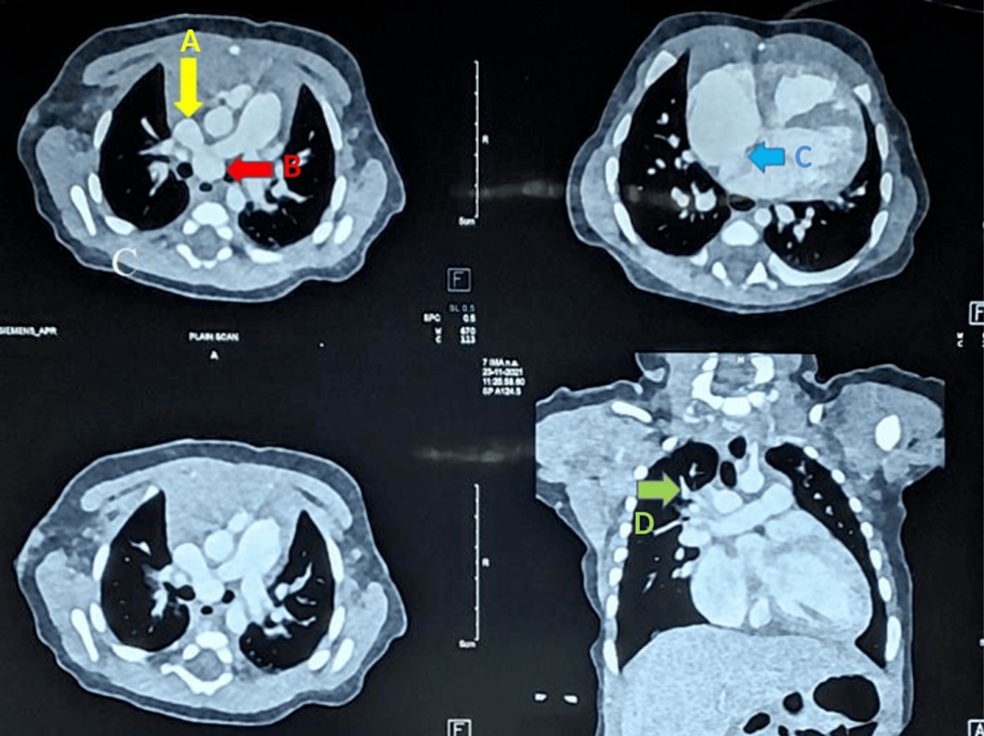
Contrast-enhanced CT angiography thorax in axial section showing common tubular vascular channel (arrow A) draining into superior vena cava (arrow B). Arrow C shows dilated right atrium and right ventricle with a small atrial septal defect. Arrow D shows pulmonary veins draining into the common tubular vascular channel. Overall findings are suggestive of supracardiac total anomalous pulmonary venous drainage with the atrial septal defect.

## Discussion

Neonates having congenital cardiac anomalies with undiagnosed complex cardiac physiology along with difficult airways coming to the CT suite on a day-care basis pose numerous challenges to the attending anaesthetist. Anaesthesia management of a neonate is demanding even for experienced anaesthetists in terms of vascular access, airway management, relatively poor respiratory reservoir and transitional cardiac physiology [3]. Neonate having cyanotic CHD with unknown cardiac physiology demands further attention. Children having CHD are associated with various airway anomalies such as bronchomalacia, laryngomalacia, stridor, subglottic stenosis, pharyngeal collapse, vocal cord paralysis, recurrent pneumonia, choanal stenosis, bronchial stenosis, tracheal stenosis, extrinsic compression of lower airways by dilated pulmonary arteries with/without left atrial dilation, dilated aorta or anomalous aortic or pulmonary arterial course [8-10] In children having CHD with coexistent airway anomaly, difficult intubation remains a possibility [9]. So proper evaluation needs to be done preoperatively to find the cause of stridor in these patients so that appropriate planning can be done for intraoperative and postoperative airway management. Children having CHD are at higher risk of developing complications such as arrhythmias, ectopics, hypoxia and cardiac arrest [11,12]. In children with CHD who develop unexpected perioperative hypoxia, differential diagnosis of reversal of shunt, pulmonary hypertensive crisis, low systemic vascular resistance (SVR) and high pulmonary vascular resistance (PVR) should be kept [11] In balanced circulation, flow to the systemic or pulmonary vasculature depends upon the relative SVR and PVR [11,12]. Most of the anaesthetic induction agents and volatile agents reduce the SVR except ketamine [11]. Changes in the ventilation and gas exchange influence the PVR. Factors, such as acidosis, hypercarbia, hypoxia, hyperinflation and raised haematocrit, increase the PVR [11-13]. Recently, there has been concern regarding the safety of anaesthetic agents in children less than three years as there is an increased risk of learning disability, attention deficit hyperactive disorder and developmental delay [4-7]. So, techniques are being developed to promote immobilisation without using anaesthesia in short procedures. One such technique is the “feed and wrap” technique where feeding and swaddling induce natural sleep in infants [14,15]. This technique is also named feed and swaddle, feed and sleep or feed and bundle [14]. In this technique, a pacifier with or without sucrose is used to soothe the baby and induce natural sleep. The infant is tightly wrapped in a swaddling blanket with the arms tucked inside and feet easily accessible to place a pulse oximeter. During the procedure, ECG, pulse oximetry and temperature monitoring can be done. With this technique, children having congenital cardiac anomalies along with difficult airways can be managed successfully with no adverse complications associated with the use of anaesthetic agents such as respiratory distress, shunt reversal, failed intubation, learning disability or developmental delay. Along with this, the feed and wrap technique does not lead to deterioration of image quality when properly used.

## Conclusions

In conclusion, the use of the feed and wrap technique should be encouraged especially for NORA with an added advantage in neonates having congenital cardiac defects with co-existent airway problems. It avoids the use of any anaesthetic agent thus avoiding hemodynamic changes, airway loss and its post-procedure residual effect. There is no need for postoperative anaesthesia care and feeding can be started as soon as possible. If the concerned staff is adequately trained, the use of this technique can minimise post-anaesthesia related complications, especially in places such as CT/MRI suites where resources are scarce to handle complications without compromising image quality.
